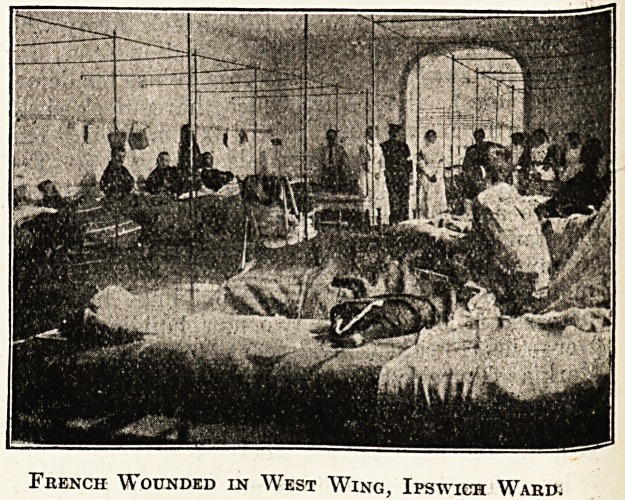# British Wounded in a French Hospital at St. Malo

**Published:** 1914-11-21

**Authors:** 


### British Wounded in a French Hospital at St. Malo.

A BRIEF record and some photographs of the interior of a girls' school, "Moka," in St. Malo, taken over by the French authorities as Supplementary Military Hospital No. 62, and now running under the auspices of the St. John Ambulance Association, may be of interest to readers in England. "Moka" contains 100 beds, and has rooms for fourteen officers. It is the only hospital in these parts which has any British wounded and sick in it. Besides the English, of whom there are nineteen, there are at present in "Moka" 31 French and 36 Belgians.

BRITISH WOUNDED IN EAST WING, IPSWICH WARD.

BRITISH SICK IN KING GEORGE V. WARD.

THEATRE, "MOKA" HOSPITAL.

FRENCH WOUNDED IN WEST WING, IPSWICH WARD.

It will be recalled that in our issue of October 31 our Special Correspondent contributed an account of "the Ipswich Ward in a new military hospital at St. Malo," of which one of the honorary staff of the East Suffolk and Ipswich Hospital, Dr. J. Hossack, was placed in charge. In the light of that account the above photographs are of exceptional interest.

## Figures and Tables

**Figure f1:**
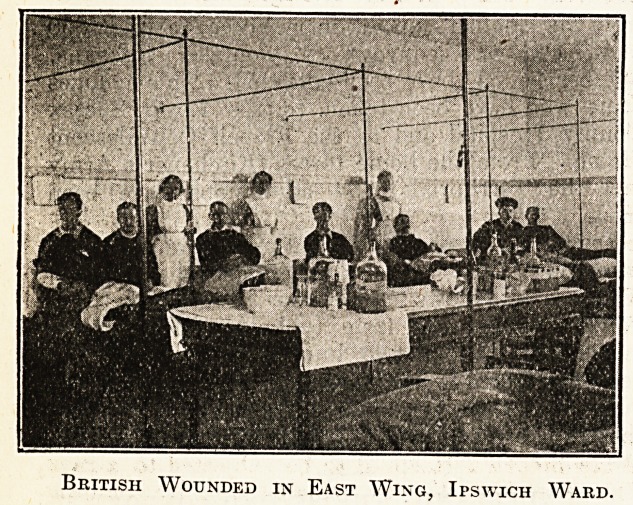


**Figure f2:**
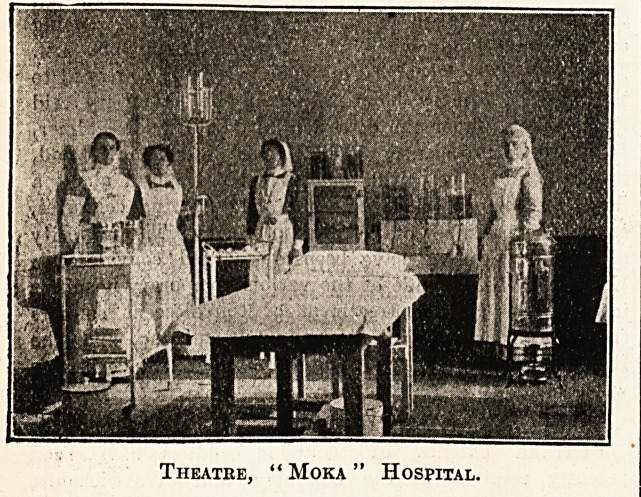


**Figure f3:**
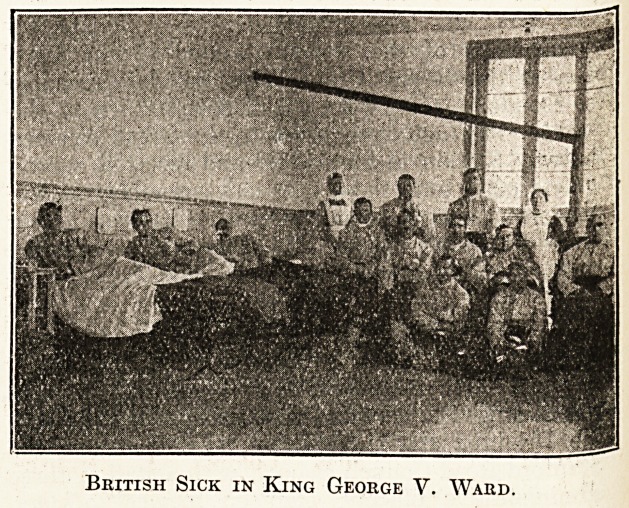


**Figure f4:**